# Utilizing Joint Routing and Capacity Assignment Algorithms to Achieve Inter- and Intra-Group Delay Fairness in Multi-Rate Multicast Wireless Sensor Networks

**DOI:** 10.3390/s130303588

**Published:** 2013-03-14

**Authors:** Frank Yeong-Sung Lin, Chiu-Han Hsiao, Leo Shih-Chang Lin, Yean-Fu Wen

**Affiliations:** 1 Department of Information Management, National Taiwan University, No. 1 Sec. 4, Roosevelt Rd., Taipei City 106, Taiwan; E-Mails: yslin@im.ntu.edu.tw (F.Y.S.L.); d98725001@ntu.edu.tw (L.S.C.L.); 2 Graduate Institute of Information Management, National Taipei University, No. 151, University Rd., San Shia District, New Taipei City 23741, Taiwan; E-Mail: yeanfu@mail.ntpu.edu.tw

**Keywords:** wireless sensor networks, multicast, end-to-end fairness, quality of service, Lagrangean Relaxation method, optimization

## Abstract

Recent advance in wireless sensor network (WSN) applications such as the Internet of Things (IoT) have attracted a lot of attention. Sensor nodes have to monitor and cooperatively pass their data, such as temperature, sound, pressure, *etc.* through the network under constrained physical or environmental conditions. The Quality of Service (QoS) is very sensitive to network delays. When resources are constrained and when the number of receivers increases rapidly, how the sensor network can provide good QoS (measured as end-to-end delay) becomes a very critical problem. In this paper; a solution to the wireless sensor network multicasting problem is proposed in which a mathematical model that provides services to accommodate delay fairness for each subscriber is constructed. Granting equal consideration to both network link capacity assignment and routing strategies for each multicast group guarantees the intra-group and inter-group delay fairness of end-to-end delay. Minimizing delay and achieving fairness is ultimately achieved through the Lagrangean Relaxation method and Subgradient Optimization Technique. Test results indicate that the new system runs with greater effectiveness and efficiency.

## 1. Introduction

**Figure 1.**
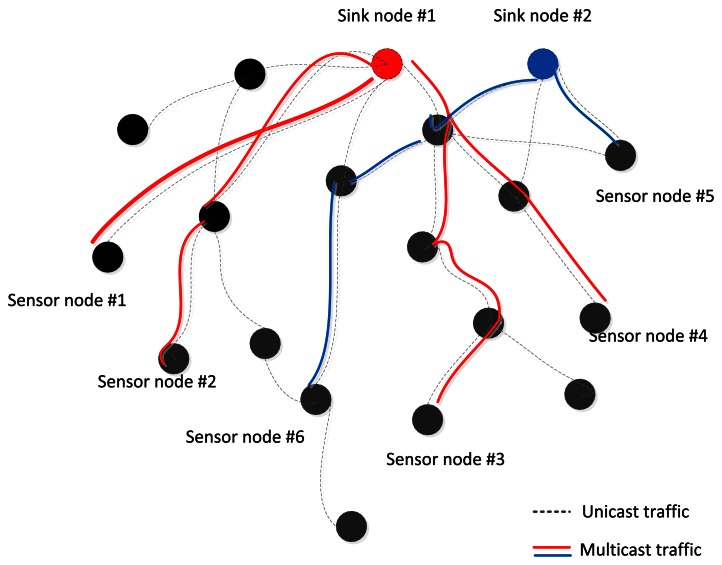
Scenario of unicast and multicast.

Wireless Sensor Network (WSN) applications such as the Internet of Things (IoT) are an integral part of the Future Internet that could be defined as a dynamic global network infrastructure with self-organization capabilities that is seamlessly integrated into the information network based on standard or interoperable communication protocols. This attracts more attention to how sensor nodes can monitor and cooperatively pass their data like temperature, sound, pressure, *etc.* more efficiently through the network under realistic physical or environmental conditions. For example: the environment could be a scale free data sampling area like groups of lakes, high mountains with original forest, or the environment is so inaccessible that humans can't get the sampling data in the short term. A survey shows that a few networks only have one existing sink node, called a tree, but other multi-sink networks exist in some specific applications, called a forest, which are more indispensable in WSNs. Figure 1 shows a scenario illustrating that common unicast routing protocols will set up separated routes from sink node #1, 2 to sensor node #1∼6, denoted by the dashed lines, but it is obvious that if multicast technology could be used, it would be a better, more energy-efficient way to suppress the duplicate transmissions of same data packets and another nodes which are not included in multicast groups could be put into power saving mode, indicated by the real lines.

Multicasting is an automatic communication technique in which data from source nodes are transmitted to a larger number of subscribed destination nodes for the purpose of networking these kinds of applications. However, maintaining a functional level of Quality-of-Service (QoS) in a multicast environment, especially in a resource-constrained wireless sensor network, can be difficult. Contemporary research indicates that this problem can be abstractly constructed and modeled through a multicast tree (forest) model with resource allocations and routing assignment problems [1,2].

In order to design a multicast sensor system and solve the problems mentioned above, the solution planning is concerned with the economic feasibility of establishing a network in the first stage, the QoS, and the fairness to subscribers in the second stage. This work can be constructed by a cost of a multicast sensing tree to determine the total resource utility of networks for multimedia applications with regards to delay in transmission. QoS metrics is a crucial for the measurement of latency, delay jitter, delay variance, and end-to-end delay per source-destination path. Lastly, we must keep in mind that subscribers may have different service level agreements in a real operation system. We also consider fairness, which measures whether resources are being adequately and fairly allocated to subscribers from both a user perspective and an operations perspective.

## 2. Literature Survey

### 2.1. IP Multicast

IP multicast is a bandwidth-conserving technology that runs the TCP/IP suite of protocols, specifically designed to reduce traffic to forward IP datagrams to members in multicast groups by simultaneously delivering a single stream of information to potentially thousands of corporate recipients. It can deliver both data and video streaming to specific users in groups, and can do so based on the structure of the multicast tree and whether it incorporates source-based routing, center-based routing, or a hybrid of the two [3]. By replacing copies for all recipients with the delivery of a single stream of information, IP Multicast is able to minimize the burden on both sending and receiving hosts and reduce overall network traffic [4]. Various structures, including Distance Vector Multicast Routing Protocol (DVMRP), Multicast Open Shortest Path First (MOSPF), Protocol-Independent Multicast Sparse Mode (PIM-SM), PIM Dense Mode (PIMDM), Core-Based Trees (CBT), Ordered CBT (OCBT), and Border Gateway Multicast Protocol (BGMP), are briefly introduced as follows [5,6].

Distance Vector Multicast Routing Protocol (DVMRP) is defined in RFC 1,075 and is used to share information between routers to facilitate the transportation of IP Multicast packets among networks. The protocol is based on the RIP protocol for forwarding packets: the router generates a routing table with the multicast group corresponding to number of devices/routers between the router and the destination.

Multicast Open Shortest Path First (MOSPF) is an extension to the Open Shortest Path First (OSPF) protocol to support multicast routing. It is allowed for routers to share information about group memberships.

PIM Sparse Mode (PIM-SM) is one of the variants of Protocol-Independent Multicast (PIM). PIM provides one-to-many and many-to-many distribution of data over Internet. It is defined in RFC 4601 and termed protocol-independent because topology discovery mechanism is not included in PIM, but instead uses routing information supplied by other traditional routing protocols such as the Routing Information Protocol (RIP), Open Shortest Path First (OSPF), Border Gateway Protocol (BGP) and Multicast Source Discovery Protocol (MSDP). PIM-SM explicitly is built unidirectional shared trees rooted at a rendezvous point per group, and optionally creates shortest-path trees per source. PIM-SM generally scales fairly well for wide-area usage.

Core-Based Trees (CBT) was proposed for making IP Multicast scalable by constructing a tree of routers. The differentiation with other protocols for multicasting is called the routing tree that comprises multiple “cores” (also known as “centres”). The core router locations are statically configured. Other routers are added by growing “branches” of a tree, comprising a chain of routers, from the core routers out towards the routers directly adjacent to the multicast group members.

The CBT protocol can form loops during periods of routing instability, and that it can consistently fail to build a connected multicast tree when the underlying routing is stable, so the Ordered CBT (OCBT) is used. The OCBT protocol is proven to eliminate these deficiencies and reduces the latency of tree repair following a link or core failure. OCBT builds a shared multicast tree distributed per group. It is suited to inter- and intra-domain multicast routing. It uses the property to guarantee that no transient or permanent loops ever form in the structure of the tree. The protocol is that routing-table loops occur in the underlying routing protocols. OCBT also improves scalability by allowing flexible placement of the cores that serve as points of connection to a multicast tree.

Border Gateway Multicast Protocol (BGMP) is a scalable multicast routing protocol which addresses how to choose a global root for a delivery tree. However, the root is a domain, not a single router, so if there is any path available to the domain connectivity can be maintained. BGMP builds a bidirectional, shared tree of domains. BGMP is used as the inter-domain or external protocol, while domains can run any multicast IGP internally (such as CBT or PIM Sparse Mode), and can build source-specific shortest-path distribution branches to supplant the shared tree where needed.

Each approach not only discerns between various structures (centralized or distributed), but also can be designed to support dense or sparse modes. The IP Multicast solutions offer benefits relating to the conservation of network bandwidth. In the case of a high-bandwidth application, such as MPEG video, IP Multicast can benefit situations with only a few receivers because a few video streams would otherwise consume a large portion of the available network bandwidth. Even for low-bandwidth applications, IP Multicast conserves resources when transmissions involve thousands of receivers like in sensor networks.

### 2.2. QoS Routing

Currently there are a lot of multimedia applications and traffic has grown significantly, so the QoS performance in routing becomes more and more important for a multicast sensor network. The routing protocols such as OSPF (Open Shortest Path First) which is created by the Dijkstra algorithm is widely used in network routing protocols for computing a routing table inside a sub-network to get a shortest transmission path [7,8].

According to [6], the QoS requirements in routing can be classified into two categories:

- Link constraints: the restrictions on the use of link to form a routing tree, such as bandwidth, link capacity, or buffer of each link.
- Path constraints (or tree constraints): the restrictions in the perspectives of the whole multicast tree. For example, the end-to-end delay from source to destination.

The goal of QoS routing is to find a feasible path (tree) with sufficient available resources to address the QoS requirements for sensor nodes in a wireless sensor network [3], as well as to achieve as much efficiency in resource utilization as possible.

Delay, bandwidth, delay jitter, throughput, or packet loss ratio are the QoS measurements of a routing strategy of a network link. In addition, the cost of a link in the multicast tree can be defined in dollars or as a function of the buffer or bandwidth utilization. In previous research, determining the available feasible paths of the optimization problem and finding the lowest-cost feasible solution is also considered. Chen and Nahrstedt have conducted a survey of various QoS routing algorithms; these can be divided into three broad classes: (1) source routing algorithms, (2) distributed routing algorithms, and (3) hierarchical routing algorithms. Chen proposes a QoS-Aware Multicast Routing Protocol (QMRP) for non-additive metrics in which he wishes to discover a feasible path with enough requested link bandwidth and buffer space management [9]. In [10], Khadivi, Samavi, and Todd introduce new single mixed metrics for multi-constraint routing. In order to adequately reduce routing complexity, QoS routing may discard some potentially useful information in the process. Nevertheless, from an operational standpoint, those mentioned above are usually taken into account.

### 2.3. Fairness

**Figure 2.**
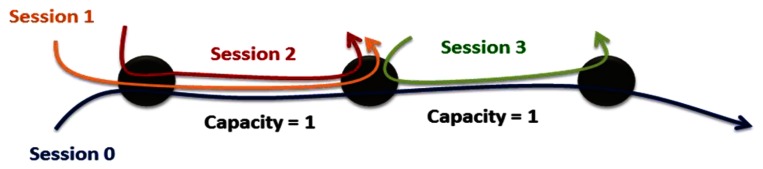
Max-min fairness.

Past research has dealt with how to optimally allocate limited resources through maximizing utility under various constraints. Limited research, however, has considered fairness in a live communication network environment. In [11], “fairness” has three definitions: max-min fairness, proportional fairness and balanced fairness. These three criteria evaluate fairness based on the channel conditions of various subscribers. The max-min fairness concept proposed by Kleinberg, Rabani and Tardos [12] incorporates the selection of routing paths, the allocation of bandwidth and the improvement of system utilities. In short, achieving max-min fairness means optimally allocating resources under the worst conditions until the system utilities are specified. From [13], Max-min fairness can be defined as: “A rate *r* is said to be *max-min fair* if it is feasible, and for each session *p ∈ P*, the allocated rate for session *p*, *r_p_*, cannot be increased while maintaining feasibility without decreasing *r_p_*_′_ for some session *p*′ for which *r_p_*_′_≤ *r_p_*”. Figure 2 can illustrates the max-min fairness, which means the max-min fairness means maximizing the allocation for the most poorly treated of those other sessions, and so forth, until all allocations are specified. The summation of traffic Session 0, 1, and 2 will be approached to the link capacity and each session has the same QoS conditions.

Max-min fairness is a way to maximize the total throughput considering the optimal fairness. Sometimes it can strike a balance between fairness and throughput by adopting the proportional fairness [14]. Proportional fairness is a compromise between fairness and throughput. It tries to maximize total throughput (but might not be the maximal throughput), while at the same time allowing all users at least the same level of service in the network [15].

### 2.4. Minimum Spanning Tree

**Figure 3.**
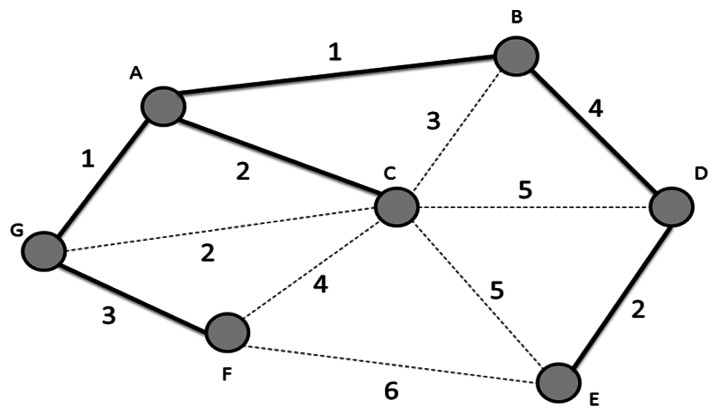
Minimum spanning tree.

In [16,17], Bazlamaçcı and Hindi propose a definition of minimum spanning tree (MST; or minimum weight spanning tree which is shown as Figure 3. MST and minimum weight spanning tree involve locating an undirected spanning tree in which the sum of the weights of the selected edges is minimum. Most works often use a simple incremental and greedy method to solve MST problems. In a greed method, the MST is built edge by edge until the best possible edge is chosen for inclusion in the MST without cycles or disconnecting in the sub-graph. The theoretical and algorithmic performance vales are compared and observed to determine the effects of network size changes. The theoretical bounds are determined by the development of an efficient MST algorithm. However, in 2002, Pettie and Ramachandran [17] proposed an optimal Minimum Spanning Forest (MSF) algorithm composed of multiple minimum spanning trees. The complexity is equal to its decision-tree complexity, *O(T* × *(m, n))*, where there are *n* nodes and *m* edges. The algorithm runs in linear time with high probability for all possible edge-weights on random graphs. The time bound ultimately depends on the edge-weight needed to determine the MSF [18]. The remaining problems of the algorithm concern the determination of worst-case complexity.

When MST is used in networks, it is necessary to consider QoS issues. Similar to the shortest path problem, when QoS constraints, such as delay, are added to the routing tree, the MST problem becomes an NP-hard problem, too. Some previous research introduces efficient heuristic algorithms to solve the problem. Salama, Reeves, and Viniotis [19] resemble Prim's algorithm which can solve the MST problem in polynomial time to formulate the problem of constructing broadcast trees for real-time traffic with delay constraints as a delay-constrained minimum spanning tree (DCMST) problem. They propose a delay-constrained minimum Steiner tree heuristic. In the comparison of the experimental result, the proposed heuristic has better performance than the existing fastest and most efficient delay-constrained minimum Steiner tree heuristic. However, in many application, multicast is more applicable.

### 2.5. Motivation

Multicast applications must fulfill a variety of requirements including bandwidth, delay, throughput, and packet loss rate. These include QoS issues regarding how to allocate constrained resources to maximize the user experience. Previous solutions to this problem might have involved constructing a multicast tree to achieve the desirable aim, but these approaches did not optimally address requirements and satisfy the needs of individual subscribers in different groups. Delay is the most important QoS metric and it is especially sensitive in a wireless communication environment. Thus, if wireless sensor networks efficiently distributes resources, wireless users should be able to access multimedia content. Subscribers in the same group may utilize the same backhaul but may have different channel conditions depending on how far or the number of hops to source node. Thus, their experiences as users may vary drastically. It is therefore important to fairly allocate resources to each connection in a multicast tree according to the end-to-end delay. According to our research, in order to achieve both routing and load balancing in the context of a non-splittable flow. The approximation algorithms proposed select the fairest possible routing path in the most optimal manner. The next would be max-min fairness to maximize the total throughput while maintaining optimal fairness.

### 2.6. Paper Organization

The rest of this paper is organized as follows: Section 3 describes the problem in a detailed and concise manner, and also includes a mathematical programming model. Section 4 presents the solution approaches for our model and develops a heuristic to get a primal feasible solution. Section 5 illustrates the simulation environment and experimental results of our approach. Finally, we present our conclusions and determine the direction of future research in Sections 6 and 7.

## 3. Problem Formulation

### 3.1. Problem Description

**Figure 4.**
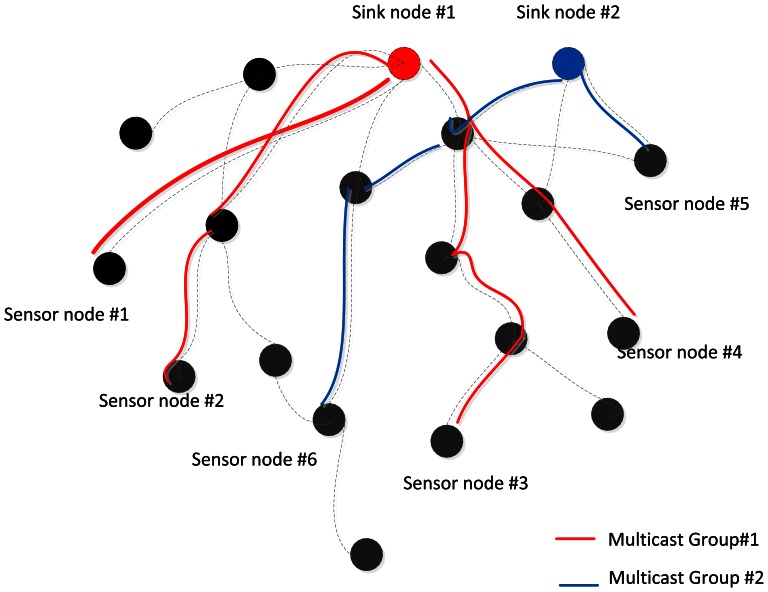
Multi-rate multicast wireless sensor network.

For the purposes of system modeling, a multicast system can be modeled as a tree. Sink node can send messages to receivers in a multicast group within the multicast tree is predefined. The sender can be viewed as a root and receivers can be viewed as leaves. Cost depends on the size and scalability of the tree, which itself depends on whether it is a single multicast tree or multiple multicast trees. Figure 4 is an example of a common case: two groups in a multicast sensor network. For each group, the root is the sink node of the multicast tree. Some sensor nodes of the multicast tree stand for the receivers in the multicast group, and others are used to forward data.

In an operation sensor network, a multi-rate multicast wireless sensor network is considered. This means each sensor node can request different quality data streams. The sink node may encode these different requirements into several different layered streams for subscribers through single or multiple multicast trees. Each source represents a distinct group, which has its own end-to-end delay.

However, most multiple-multicast routing problems of their objectives focus on the way of finding a set of routing trees satisfied with constraints. One of these constraints is limited bandwidth. The link capacity of the wireless sensor network might be contented by how many sensor nodes are existed and the traffic sessions are created in the routing paths of the multicast groups. Briefly, our model is designed to determine the following: (1) what multiple-multicast-group routing strategies are optimal; (2) how much capacity is allocated to the selected links used in the multicast sensor network; (3) what is the minimum end-to-end intra-delay per path in a multicast group; (4) How do various approaches towards achieving the minimum end-to-end inter-group experience delay among different multicast groups.

**Figure 5.**
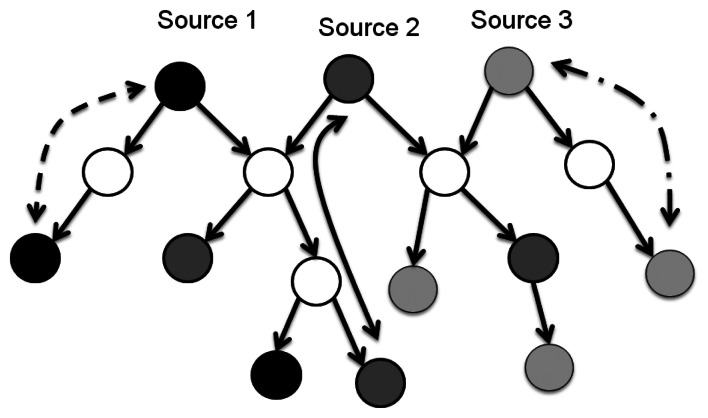
Multicast sensor networks.

In the model which is shown in Figure 5, we make assumptions regarding to consider the delay fairness to find the minimum end-to-end delay while dealing with constrains of a multicast WSN. Based on the purpose, listed below are our assumptions, givens, and objectives:

**Assumptions:**

All routers are stationary.

Each link is adopted with a M/M/1 queuing model. The delay function d = 1/(C − F) subject to C > F(C means the capacity on a link, F means the aggregation flow).

The aggregation flow is always less than the capacity on a link, otherwise the congestion occurs results in system crashed. When the capacity is given, the aggregated flow is finite and less than the given capacity, otherwise the extra flow has to transmit through other links by routing assignments. Once the capacity is given as a constant, the buffer size of each router does not required to be infinite. Just set an enough size, say ≥link capacity, to handle the buffer requirement. In this way, the backbone capacity is also large enough to handle the traffic. Because (1) bandwidth of the sensor network is small and much less than the backbone fiber capacity, (2) the range to calculate the end-to-end delay is set from source to sink node, the do not need to consider the backbone capacity, (3) the backbone capacity is larger than the sensor network, the backbone link will not cause congestion or delay on the wireless sensor network.

**Given:**

The set of all nodes in the network.

The set of all links in the network.

The set of multicast source nodes.

The set of destinations for each source.

The set of paths from each source to it destinations.

The discrete traffic requirements for subscribers in multicast groups.

The degree of importance of each multicast group.

The minimum hop counts from the farthest destination node on each multicast group.

**Objective:**

To minimize the end-to-end inter-delay among multicast groups.

**Subject to:**

The flow of each link is limited by its allocated link capacity and the maximum traffic of its all sublinks.

All of the selected paths in a group will form a multicast tree.

The total number of links in a multicast tree is the biggest numbers of the numbers in minimum hops of farthest destinations between the numbers of destinations.

Delay constraints include intra-group and inter-group end-to-end delay. Inter-group end-to-end delay per path should be consistent within a multicast group. Additionally, inter-group end-to-end delay among multicast groups should be equal when considering the weight of each group.

**To determine:**

A multiple-multicast-group network for each source to reach their destinations respectively.

The capacity allocated to the selected links used in the multicast network.

The minimal end-to-end intra-delay per path in a multicast group.The minimal end-to-end inter-group delay among different multicast groups.

### 3.2. Mathematical Formulation

The aforementioned problem can be modeled through mathematical programming. The followings are parameters and decision variables; corresponding notations are defined in Tables 1 and 2.

**Objective Function:**

(IP 1) $$Z_{IP1}=\underset{s\in S}{\min}T,$$

**Subject to:**

**Delay Constraints**

(1.1) $$\begin{array}{ll} \beta_{s}t_{s}=T & \forall s\in S \end{array}$$

(1.2) $$\begin{array}{ll} \sum_{l\in L}h_{\text{sdl}}D_{sl}(c_{sl},g_{sl})=t_{s} & \forall s\in S,d\in D_{s} \end{array}$$

**Routing Constraints**

(1.3) $$\begin{array}{ll} \sum_{p\in P_{sd}}x_{p}\delta_{pl}=h_{\text{sdl}} & \forall s\in S,\forall d\in D_{s},l\in L \end{array}$$

(1.4) $$\begin{array}{ll} \sum_{p\in P_{sd}}x_{p}=1 & \forall s\in S,d\in D_{s} \end{array}$$

(1.5) $$\begin{array}{ll} \sum_{d\in D_{s}}h_{\text{sdl}}\leq \left|D_{s}\right|y_{sl} & \forall s\in S,l\in L \end{array}$$

**Capacity and Traffic Constraints**

(1.6) $$\begin{array}{ll} g_{sl}=h_{\text{sdl}}\gamma_{sd}<c_{sl} & \forall s\in S,\forall d\in D_{s},l\in L \end{array}$$

(1.7) $$\begin{array}{ll} g_{sl}\in [0,\underset{d\in D_{s}}{\max}r_{sd}] & \forall s\in S,l\in L \end{array}$$

(1.8) $$\begin{array}{ll} \sum_{s\in S}c_{sl}\leq c_{l} & \forall l\in L \end{array}$$

(1.9) $$\begin{array}{ll} \sum_{l\in L_{v}}c_{l}\leq C_{v} & \forall v\in V \end{array}$$

**Tree Constraints**

(1.10) $$\begin{array}{ll} \sum_{l\in L}y_{sl}\geq \max\left\{H_{s},\left|D_{s}\right|\right\} & \forall s\in S \end{array}$$

(1.11) $$\begin{array}{ll} \sum_{l\in I_{v}}y_{sl}\leq 1 & \forall s\in S,v\in V-\{S\} \end{array}$$

(1.12) $$\begin{array}{ll} \sum_{l\in I_{s}}y_{sl}=0 & \forall s\in S \end{array}$$

**Integer Constraints**

(1.13) $$\begin{array}{ll} x_{p}=0\;\text{or}\;1 & \forall s\in S,p\in P_{sd},d\in D_{s} \end{array}$$

(1.14) $$\begin{array}{ll} y_{sl}=0\;\text{or}\;1 & \forall s\in S,l\in L \end{array}$$

(1.15) $$\begin{array}{ll} h_{\text{sdl}}=0\;\text{or}\;1 & \forall s\in S,\forall d\in D_{s},l\in L. \end{array}$$

Explanation of Objective Function:

The objective function (IP 1) is to minimize the end-to-end inter-delay in group *T*. The group *T* is obtained by multiplying any end-to-end intra-delay *t_s_* corresponding to the group weight *β_s_*. Therefore, *T* is also restricted both by the intra-group and inter-group end-to-end delay fairness.

Explanation of Constraints:

Constraint (1.1) confines the end-to-end inter-delay fairness among groups. In (1.1), taking the given weights of all groups into account, we multiply the end-to-end intra-delay for each group by the given weights *β_s_*. The result should be equal to the inter-delay decision variable, T. Thus, we can confirm 100% inter-delay fairness in the whole multiple-multicast wireless sensor network.

- Constraint (1.2) confines the intra-group end-to-end delay fairness. Considering the to-be-determined link capacity, *c_sl_*, and the traffic flow, *g_sl_*, for a link *l* belonging to multicast group *s*, we can obtain the minimal end-to-end delay among each path, which should be equal to the intra-delay variable *t_s_* on the right side of the equation, thus achieving 100% intra-delay fairness.
- An auxiliary variable, *t_sdl_*, is used to represent the relationship between link *l* and path *p* for multicast group *s*. That is, if link *l* is selected by destination *d* in multicast group *s*, making *t_sdl_* equal to 1, it must also be on the path adopted by destination d in multicast group *s*.
- Constraint (1.4) means that only one path can be selected for a destination *d* belonging to a multicast group *s*.
- Constraint (1.5) confirms that if one path is selected for destination *d* belonging to multicast group *s*, it must also be on the subtree adopted by multicast group *s*. For example, if a link is selected for two different destinations in a multicast group, the left side of Constraint (1.5) should be 2. The right side of Constraint (1.5), is therefore used to make sure that the selected link must also be selected by *y_sl_* at most for all destinations in the multicast group, which is equal to the right side of Constraint (1.5).
- The other auxiliary variable, *g_sl_*, is used to stand for the collocated traffic flow in the link *l* for multicast group *s*. Therefore, Constraint (1.6) ensures that the maximal traffic flow passing through the link *l* in group *s* does not exceed the link capacity allocated for link *l* in multicast group *s*.
- Constraint (1.7) gives a discrete range of traffic rates for each *g_sl_*, which is from 0 to the maximal bandwidth requested by a destination *d*, which belongs to multicast group *s*.
- Constraint (1.8) confines that for each link *l*, the sum of the allocated capacity on the link *l* among all multicast groups does not exceed the link's allocated capacities *c_l_*.
- Constraint (1.9) confines that for each node *v*, sum of the allocated capacity of all out-coming link *l* from node *v* does not exceed the node's physical capacities *C_v_*. Constraint (1.8) and Constraint (1.9) also shows the contenting relationship among groups in wireless sensor network.
- Constraint (1.10) confines that the total number of links in the multicast tree rooted at source s is at least the maximal value chosen from the height of the multicast tree, *H_s_*, and the number of destinations *D_s_*. It also ensures that the number of all the links in a multicast tree *s* should exceed the number of the destinations.
- Constraints (1.11) and (1.12) are both redundant constraints. Constraint (1.11) requires the number of selected incoming links *y_sl_* to node is 1 or 0. Constraint (1.12) requires that there is no selected incoming links *y_sl_* to node that is the source of multicast group *s*.
- Constraints (1.13), (1.14) and (1.15) are the integer constraints.

### 3.3. Introduction to Lagrangean Relaxation Method

In mathematics and computer science researches, the so-called optimization problem is informally referred to the problem of finding the best or the optimal solution of all feasible solutions. The optimal solution usually is the minimum or maximum value, depending on the objective function subjected to constraints. For example, if the minimum solution can be found of a generic non-linear programming problem, the formulation and presentation can be defined as below:

(1) $$\begin{array}{c} \text{Minimize}:Z=f(x),\\ \text{subject to}:g_{i}(x)\leq 0,h_{j}(x)=0,\\ i=1,\ldots ,m,j=1,\ldots ,m,X\in R. \end{array}$$

**Figure 6.**
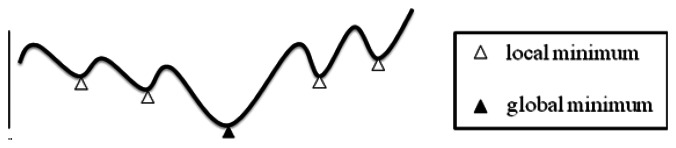
Solution of a general non-linear programming problem.

Figure 6 illustrates a generic non-linear programming problem which we want to solve. The curve of the solutions is involved many local minimum and only one global minimum. The objective would be the global minimum. In order to solve this kind of optimization problem, Lagrangean Relaxation (LR) method is a good way to calculate or get the solutions by approximation to the global optimality.

In the iteration procedures of LR, a decomposition method is usually used to divide the problem into several relatively simpler sub-problems of a complex problem. Based on well-developed algorithms, the objective could be solved easily to find local minima of these sub-problems, thus solving the primal problem and approaching the global minimum.

The Lagrangean Relaxation method had proposed since early 1970s for use in large-scale mathematical programming applications [20,21]. The Lagrangean Relaxation method is flexible and effective for solving optimization problems such as integer programming, liner programming with combinatorial objective function, or non-linear programming problems.

**Figure 7.**
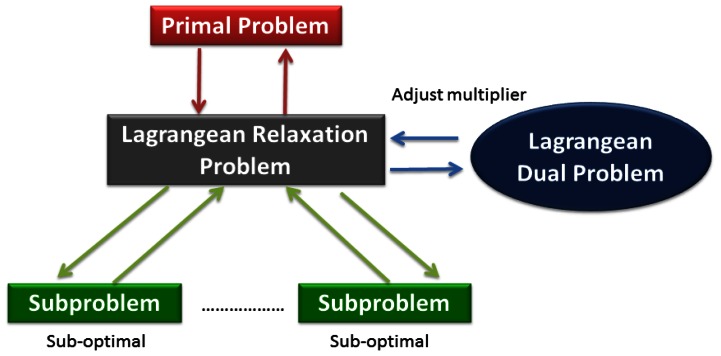
State diagram of Lagrangean Relaxation method.

The main idea of the Lagrangean Relaxation method is to pull apart the model by relaxing (*i.e.*, removing) complicated constraints in the primal optimization problem. The next procedure could be modified to the objective function corresponding to associated Lagrangean multipliers of relaxed constraints [22]. The primal optimization problem can be transformed into a Lagrangean Relaxation form. The Lagrangean Relaxation problem is separated into several independent sub-problems in each decision variables or other rules by applying the decomposition method. For sub-problems, we can design some heuristics or algorithms to apply and find the optimal value. Figure 7 illustrates the procedure state diagram of a Lagrangean Relaxation. For example, if the problem is a minimization problem, the optimal value of the relaxed constraints is always a lower bound on the optimal value of original problem under the relaxed conditions. The lower bound can be improved by adjusting the set of multipliers iteration by iteration to reduce the gap of the solution between the primal problem and the Lagrangean Relaxation. This procedure is also called the Lagrangean Dual problem.

**Figure 8.**
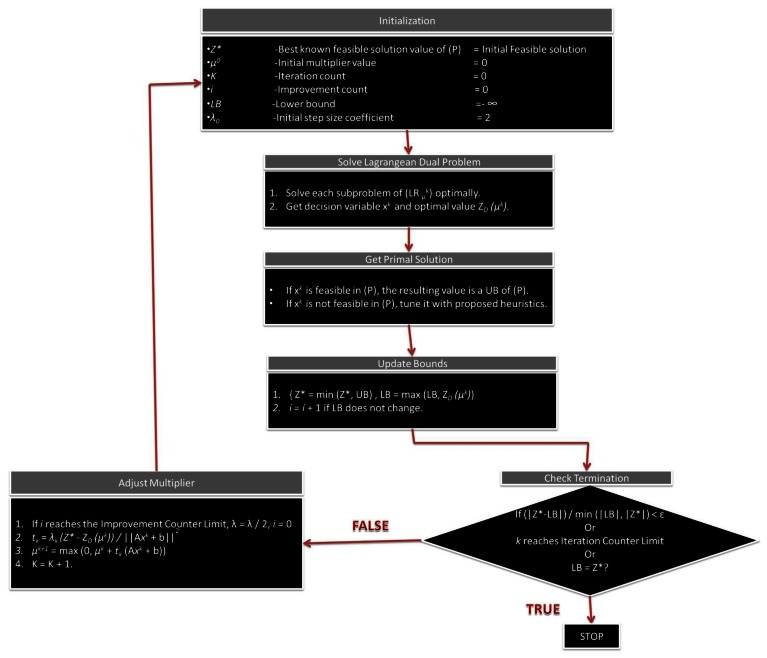
Procedures of Lagrangean Relaxation method.

If the iterations of LR processes are done, which means the optimal feasible solution from the Lagrangean Relaxation problem is determined when the constraints are satisfied. If the feasible solution in the primal problem is not satisfied by the constraints, the heuristic procedures of the iterations would be designed for tuning the infeasible solution until it becomes a feasible one. Figure 8 shows the complete Lagrangean Relaxation method procedure step by step.

## 4. Solution Approach

In this paper, one problem encountered when constructing a multicast sensor network is duly considering QoS requirements for each user and retaining fairness between them. To address this problem, two mathematical programming techniques, the Lagrangean Relaxation Method [13] and the Subgrandient Method, are adopted [16]. By utilizing the Lagrangean Relaxation Method and Subgradient Method, we can devise a feasible solution of a multicast sensor network and ultimately achieve the goals of efficiently allocating link capacity while maintaining fairness between users.

### 4.1. Objective and Constrains

By introducing Lagrangean Multiplier Vectors *μ_1_, μ_2_, μ_3_, μ_4_, μ_5_, μ_6_, μ_7_* and *μ_8_*, the primal problem can be solved through Lagrangean Relaxation. For the purpose of applying Lagrangean Relaxation, the original problem formulation (IP 1) was reformulated into an equivalent formulation (IP 2) below:

**Objective Function:**

(IP 2) $$Z_{IP1}=\underset{s\in S}{\min}T,$$

**Subject to:**

**Delay Constraints**

(2.1) $$\begin{array}{ll} \beta_{s}t_{s}=T & \forall s\in S \end{array}$$

(2.2) $$\begin{array}{ll} \sum_{l\in L}h_{\text{sdl}}D_{sl}(c_{sl},g_{sl})=t_{s} & \forall s\in S,d\in D_{s} \end{array}$$

**Routing Constraints**

(2.3) $$\begin{array}{ll} \sum_{p\in P_{sd}}x_{p}\delta_{pl}=h_{\text{sdl}} & \forall s\in S,\forall d\in D_{s},l\in L \end{array}$$

(2.4) $$\begin{array}{ll} \sum_{p\in P_{sd}}x_{p}=1 & \forall s\in S,d\in D_{s}, \end{array}$$

(2.5) $$\begin{array}{ll} \sum_{d\in D_{s}}h_{\text{sdl}}\leq \left|D_{s}\right|y_{sl} & \forall s\in S,l\in L \end{array}$$

**Capacity and Traffic Constraints**

(2.6) $$\begin{array}{ll} h_{\text{sdl}}\gamma_{sd}\leq g_{sl} & \forall s\in S,\forall d\in D_{s},l\in L \end{array}$$

(2.7) $$\begin{array}{ll} g_{sl}<c_{sl} & \forall s\in S,l\in L \end{array}$$

(2.8) $$\begin{array}{ll} g_{sl}\in [0,\underset{d\in D_{s}}{\max}r_{sd}] & \forall s\in S,l\in L \end{array}$$

(2.9) $$\begin{array}{ll} \sum_{s\in S}c_{sl}\leq c_{l} & \forall l\in L \end{array}$$

(2.10) $$\begin{array}{ll} \sum_{l\in L_{v}}c_{l}\leq C_{v} & \forall v\in V \end{array}$$

**Tree Constraints**

(2.11) $$\begin{array}{ll} \sum_{l\in L}y_{sl}\geq \max\left\{H_{s},\left|D_{s}\right|\right\} & \forall s\in S \end{array}$$

(2.12) $$\begin{array}{ll} \sum_{l\in I_{v}}y_{sl}\leq 1 & \forall s\in S,v\in V-\{S\} \end{array}$$

(2.13) $$\begin{array}{ll} \sum_{l\in I_{s}}y_{sl}=0 & \forall s\in S \end{array}$$

**Integer Constraints**

(2.14) $$\begin{array}{ll} x_{p}=0\;\text{or}\;1 & \forall s\in S,p\in P_{sd},d\in D_{s} \end{array}$$

(2.15) $$\begin{array}{ll} y_{sl}=0\;\text{or}\;1 & \forall s\in S,l\in L \end{array}$$

(2.16) $$\begin{array}{ll} h_{\text{sdl}}=0\;\text{or}\;1 & \forall s\in S,\forall d\in D_{s},l\in L. \end{array}$$

Amended constraints are added in (2.6) and (2.7). Constraints (2.1), (2.2), (2.3), (2.5), (2.6), (2.7), (2.9) and (2.10) in (IP 2) are relaxed and multiplied by nonnegative Lagrangean multiplier vectors respectively. The LR objective function can be obtained as following:

**Optimization Problem (LR):**

(LR 1) $$\begin{array}{l} {\text{Z}}_{\text{LR}}(\mu_{\text{sdl}}^{1},\mu_{sl}^{2},\mu_{\text{sdl}}^{3},\mu_{l}^{4},\mu_{sd}^{5},\mu_{s}^{6},\mu_{sl}^{7},\mu_{v}^{8})=\\ \min T\\ +\sum_{s\in S}\sum_{d\in D_{s}}\sum_{l\in L}\mu_{\text{sdl}}^{1}(\sum_{p\in P_{sd}}x_{p}\delta_{pl}-h_{\text{sdl}})\\ +\sum_{s\in S}\sum_{l\in L}\mu_{sl}^{2}(\sum_{d\in D_{s}}h_{\text{sdl}}-\left|D_{s}\right|y_{sl})\\ +\sum_{s\in S}\sum_{d\in D_{s}}\sum_{l\in L}\mu_{\text{sdl}}^{3}(h_{\text{sdl}}r_{sd}-g_{sl})\\ +\sum_{l\in L}\mu_{l}^{4}(\sum_{s\in S}c_{sl}-c_{l})\\ +\sum_{s\in S}\sum_{d\in D_{s}}\mu_{sd}^{5}(\sum_{l\in L}h_{\text{sdl}}D_{sl}(c_{sl},g_{sl})-t_{s})\\ +\sum_{s\in S}\mu_{s}^{6}(\beta_{s}t_{s}-T)\\ +\sum_{s\in S}\sum_{l\in L}\mu_{sl}^{7}(g_{sl}-c_{sl})\\ +\sum_{v\in V}\mu_{v}^{8}(\sum_{l\in L_{v}}c_{l}-C_{v}), \end{array}$$

subject to:

(LR 1.1) $$\begin{array}{ll} x_{p}=0\;or\;1 & \forall p\in P_{sd},s\in S,d\in D_{s} \end{array}$$

(LR 1.2) $$\begin{array}{ll} y_{sl}=0\;or\;1 & \forall s\in S,l\in L \end{array}$$

(LR 1.3) $$\begin{array}{ll} h_{\text{sdl}}=0\;\text{or}\;1 & \forall s\in S,\forall d\in D_{s},l\in L \end{array}$$

(LR 1.4) $$\begin{array}{ll} \sum_{p\in P_{sd}}x_{p}=1 & \forall s\in S,d\in D_{s} \end{array}$$

(LR 1.5) $$\begin{array}{ll} g_{sl}\in [0,\underset{d\in D_{s}}{\max}r_{sd}] & \forall s\in S,l\in L \end{array}$$

(LR 1.6) $$\begin{array}{ll} \sum_{l\in L}y_{sl}\geq \max\left\{H_{s},\left|D_{s}\right|\right\} & \forall s\in S \end{array}$$

(LR 1.7) $$\begin{array}{ll} \sum_{l\in I_{v}}y_{sl}\leq 1 & \forall s\in S,v\in V-\{S\} \end{array}$$

(LR 1.8) $$\begin{array}{ll} \sum_{l\in I_{s}}y_{sl}=0. & \forall s\in S \end{array}$$

#### 4.1.1. Subproblem 1 (Related Decision Variable *x_p_*)

$${\text{Z}}_{\text{sub}1.1}(\mu_{sl}^{1})=\min\sum_{s\in S}\sum_{d\in D_{s}}\sum_{p\in P_{sd}}\sum_{l\in L}\mu_{\text{sdl}}^{1}x_{p}\delta_{pl},$$

**Subject to:**

(LR 1.1) $$\begin{array}{ll} x_{p}=0\;or\;1 & \forall p\in P_{sd},s\in S,d\in D_{s} \end{array}$$

(LR 1.4) $$\begin{array}{ll} \sum_{p\in P_{sd}}x_{p}=1 & \forall s\in S,d\in D_{s}. \end{array}$$

Subproblem 1 can be further divided into |*S*||*D_s_*| independent shortest path problems with arc weight of $\mu_{\text{sdl}}^{1}$. Each shortest path problem can be easily solved by Dijkstra's algorithm.

#### 4.1.2. Subproblem 2 (Related Decision Variable *y_sl_*)

$${\text{Z}}_{\text{sub}1.2}(\mu_{sl}^{2})=\min\sum_{s\in S}\sum_{l\in L}(-\mu_{sl}^{2}\left|D_{s}\right|)y_{sl},$$

**Subject to:**

(LR 1.2) $$\begin{array}{ll} y_{sl}=0\;or\;1 & \forall s\in S,l\in L \end{array}$$

(LR 1.6) $$\begin{array}{ll} \sum_{l\in L}y_{sl}\geq \max\left\{H_{s},\left|D_{s}\right|\right\} & \forall s\in S \end{array}$$

(LR 1.7) $$\begin{array}{ll} \sum_{l\in I_{s}}y_{sl}=0 & \forall s\in S \end{array}$$

(LR 1.8) $$\begin{array}{ll} \sum_{l\in I_{v}}y_{sl}\leq 1 & \forall s\in S,v\in V-\{S\}. \end{array}$$

Subproblem 2 can be further divided into |*S*| independent subproblems. For each multicast group *s*, here is an algorithm [2] stated as following to solve each subproblem:

**Step 1:** Compute max{*H_s_*,|*D_s_*|}for each multicast group *s*.
**Step 2:** For each group s, compute the coefficient $\left(-\mu_{sl}^{2}D_{s}|\right)$ for each link, and count the number of the coefficient $\left(-\mu_{sl}^{2}D_{s}|\right)$.
**Step 3:** If the counting number is larger than max{*H_s_*,|*D_s_*|}, assign the corresponding *y_sl_* to 1, and others are set to 0.
**Step 4:** If the counting number is not larger than max{*H_s_*,|*D_s_*|}, assign the corresponding *y_sl_* to 1. Then, assign the remaining {max{*H_s_*,|*D_s_*|}—the counting number] of smallest positive coefficients' corresponding *y_sl_* to 1, and others are set to 0.

#### 4.1.3. Subproblem 3 (Related Decision Variable *c_sl_, g_sl_* and *h_sdl_*)

$${\text{Z}}_{\text{sub}1.3}(\mu_{\text{sdl}}^{1},\mu_{sl}^{2},\mu_{\text{sdl}}^{3},\mu_{l}^{4},\mu_{sd}^{5},\mu_{sl}^{7})=\min\sum_{s\in S}\sum_{l\in L}(\sum_{d\in D_{s}}(\mu_{sl}^{2}+\mu_{\text{sdl}}^{3}r_{sd}+\mu_{sd}^{5}D_{sl}(c_{sl},g_{sl})-\mu_{\text{sdl}}^{1})h_{\text{sdl}}+(\mu_{l}^{4}-\mu_{sl}^{7})c_{sl}+(\mu_{sl}^{7}-\sum_{d\in D_{s}}\mu_{\text{sdl}}^{3})g_{sl}),$$

**Subject to:**

(LR 1.3) $$\begin{array}{ll} h_{\text{sdl}}=0\;\text{or}\;1 & \forall s\in S,\forall d\in D_{s},l\in L \end{array}$$

(LR 1.5) $$\begin{array}{ll} g_{sl}\in [0,\underset{d\in D_{s}}{\max}r_{sd}] & \forall s\in S,l\in L. \end{array}$$

Subproblem 3 can be decomposed into |*S*||*L*| independent subproblems involved a delay function. For each link *l* ∈ *L* in a multicast group *s* ∈ *S*:

$$\min\sum_{d\in D_{s}}(\mu_{sl}^{2}+\mu_{\text{sdl}}^{3}r_{sd}+\mu_{sd}^{5}D_{sl}(c_{sl},g_{sl})-\mu_{\text{sdl}}^{1})h_{\text{sdl}}+(\mu_{l}^{4}-\mu_{sl}^{7})c_{sl}+(\mu_{sl}^{7}-\sum_{d\in D_{s}}\mu_{\text{sdl}}^{3})g_{sl}.$$

**Figure 9.**
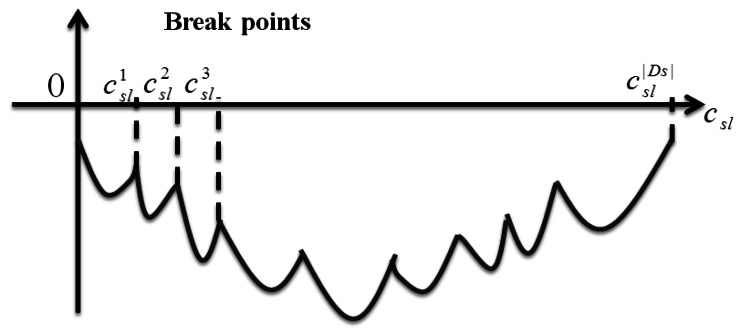
An example of Subproblem 3 considering *c_sl_*.

However, the decomposed subproblem is a complicated problem due to the coupling of *h_sdl_*, *c_sl_* and *g_sl_*. Since the auxiliary variable *g_sl_* is a discrete and finite set. The optimal solution can be computed and compared from all finite results to find out. Then *h_sdl_* and *g_sl_* are considered. According to the algorithm developed in [23], subproblems can be solved and decomposed by the following steps:

**Step 1:** First of all, the parts of decision variable *h_sdl_* can be solved by $\left(\mu_{sl}^{2}+\mu_{\text{sdl}}^{3}r_{sd}+\mu_{sd}^{5}D_{sl}(c_{sl},g_{sl})-\mu_{\text{sdl}}^{1}=0\right)$ for each destination *d* in multicast group *s*. These |*D_s_*| numbers of *c_sl_* is the so-called break points illustrated in Figure 9.
**Step 2:** Sort these breaking points and denote them as $c_{sl}^{1},c_{sl}^{2}\ldots \ldots .c_{sl}^{\left|D_{s}\right|}$ with the sequence from smallest to largest.
**Step 3:** At each interval, $c_{sl}^{i}\leq c_{sl}\leq c_{sl}^{i+1}$, the corresponding *h_sdl_* is 1 if $\mu_{sl}^{2}+\mu_{\text{sdl}}^{3}r_{sd}+\mu_{sd}^{5}D_{sl}(c_{sl},g_{sl})-\mu_{\text{sdl}}^{1}\leq 0$, otherwise *h_sdl_* is 0.
**Step 4:** Until now we only need to determine the value of *c_sl_*. We regard these |*S*||*L*| independent subproblems as an auxiliary function *θ_sl_*(*c_sl_*) denoted as following: $\theta_{sl}(c_{sl})=\underset{d\in D_{s}}{\text{∑}}(\mu_{sl}^{2}+\mu_{\text{sdl}}^{3}r_{sd}+\mu_{sd}^{5}D_{sl}(c_{sl},g_{sl})-\mu_{\text{sdl}}^{1})h_{\text{sdl}}+(\mu_{l}^{4}-\mu_{sl}^{7})c_{sl}+(\mu_{sl}^{7}-\underset{d\in D_{s}}{\text{∑}}\mu_{\text{sdl}}^{3}){\text{g}}_{\text{sl}}$. Within the interval, $c_{sl}^{i}\leq c_{sl}\leq c_{sl}^{i+1}$, we can get the minimal value of *c_sl_* by first differential. Then, let $\underset{d\in D_{s}}{\text{∑}}\mu_{sd}^{5}h_{\text{sdl}}=a_{1}$, and the local minimal *c_sl_* must be either at the smaller boundary point, $c_{sl}^{i}$ or $c_{sl}^{i+1}$, or at point $c_{sl}^{*}=g_{sl}+\sqrt{\frac{a_{1}}{\left(\mu_{l}^{4}-\mu_{sl}^{7}\right)}}$.
**Step 5:** The global minimum point can be found by comparing these |*D_s_*| local minimum points for each |*S*||*L*| iteration.

#### 4.1.4. Subproblem 4 (Related Decision Variable *t_s_*)

$${\text{Z}}_{\text{sub}1.4}(\mu_{sd}^{4},\mu_{s}^{6})=\min\underset{s\in S}{\text{∑}}(\mu_{s}^{6}\beta_{s}-\underset{d\in D_{s}}{\text{∑}}\mu_{sd}^{5})t_{s},$$

Subject to the lower and upper bound of *t_s_*.

To minimize the objective function of Subproblem 4 is determined by $\left(\mu_{s}^{6}\beta_{s}-\underset{d\in D_{s}}{\text{∑}}\mu_{sd}^{5}\right)$ for each *t_s_*. When the coefficient is negative, the upper bound of *t_s_* is iterated into the objective; otherwise the lower bound of *t_s_* is iterated. The initial lower bound of *t_s_* can be found when all traffic flows are fully distributed over different selected links. Therefore, it is best to have a destination with the smallest traffic requirement routes along a one-hop path to the destination. Therefore, the initial lower bound of *t_s_* should be *D_sl_* (*C_v_*, min *r_sd_*).

On the other hand, the initial upper bound of *t_s_* could be initialized as a worst-case scenario. The initial upper bound of *t_s_* is set when all traffic flow is completely aggregated along the longest path. So, the initial upper bound of *t_s_* should be $D_{sl}(\frac{\max r_{sd}*C_{v}}{\underset{s\in S}{\text{∑}}\left(\max r_{sd}\right)},\min(\max r_{sd}))*H_{s}$.

#### 4.1.5. Subproblem 5 (Related Decision Variable *T*)

$${\text{Z}}_{\text{sub}1.5}(\mu_{s}^{6})=\min(1-\underset{s\in S}{\text{∑}}\mu_{s}^{6})T,$$

Subject to the lower and upper bound of *T*.

To minimize the objective function of Subproblem 5 is determined by $\left(1-\underset{s\in S}{\text{∑}}\mu_{s}^{6}\right)$. When the coefficient is negative, the upper bound of *T* is iterated into the objective; otherwise the lower bound of *T* is iterated. According to Subproblem 4, we might infer that the initial lower bound of *T* should be equal to $D_{sl}(C_{v},\min(\max r_{sd}))*\min\beta_{s}$. Besides, the initial upper bound of *T* should be $(D_{sl}(\frac{\max r_{sd}*C_{v}}{\underset{s\in S}{\text{∑}}\left(\max r_{sd}\right)},\min(\max r_{sd}))*H_{s})*\max\beta_{s}$.

#### 4.1.6. Subproblem 6 (Related Decision Variable *c_l_*)

$${\text{Z}}_{\text{sub}1.6}(\mu_{l}^{4},\mu_{v}^{8})=\min(\sum_{v\in V}\sum_{l\in L_{v}}\mu_{v}^{8}-\sum_{l\in L}\mu_{l}^{4})c_{l},$$

Subject to the lower and upper bound of *c_l_*.

In Subproblem 6, because not every link *l* ∈ *L* is allocated capacity, we make the feasible link set be *l* ∈ *L_v_*. Hence, we can reformulate the object function of Subproblem 6 as $\min\sum_{l\in L_{v}}(\sum_{v\in V}\mu_{v}^{8}-\mu_{l}^{4})c_{l}$.

To minimize the objective function of Subproblem 6 is determined by $\left(\sum_{v\in V}\mu_{v}^{8}-\mu_{l}^{4}\right)$ for each *c_l_*. When the coefficient is negative, the upper bound of *c_l_* is iterated into the objective; otherwise the lower bound of *c_l_* is iterated. Therefore, the initial lower bound of *c_l_* could be the smallest *r_sd_*, and the initial upper bound of *c_l_* could be the whole node capacity, *C_v_*.

### 4.2. The Dual Problem and the Subgradient Method

According to the weak Lagrangean Duality Theorem, for any $\mu_{sl}^{2},\mu_{\text{sdl}}^{3},\mu_{l}^{4},\mu_{sl}^{7},\mu_{v}^{8}\geq 0$. *Z_D1.1_* $\left(\mu_{\text{sdl}}^{1},\mu_{sl}^{2},\mu_{\text{sdl}}^{3},\mu_{l}^{4},\mu_{sd}^{5},\mu_{s}^{6},\mu_{sl}^{7},\mu_{v}^{8}\right)$ is a lower bound on *Z_IP1_*. The following dual problem (D1) is then constructed to calculate the tightest lower bound.

Dual Problem (D1):

$$\begin{array}{c} {\text{Z}}_{\text{D}1}=\max{\text{Z}}_{\text{D}1}(\mu_{\text{sdl}}^{1},\mu_{sl}^{2},\mu_{\text{sdl}}^{3},\mu_{l}^{4},\mu_{sd}^{5},\mu_{s}^{6},\mu_{sl}^{7},\mu_{v}^{8}),\\ \text{Subject to}:\mu_{sl}^{2},\mu_{\text{sdl}}^{3},\mu_{l}^{4},\mu_{sl}^{7},\mu_{v}^{8}\geq 0. \end{array}$$

There are several methods to solve the dual problem (D1). Among them is the most popular method, the subgradient method, which is employer in [7]. Let a vector *g* be a subgradient of Z_D1_ $\left(\mu_{\text{sdl}}^{1},\mu_{sl}^{2},\mu_{\text{sdl}}^{3},\mu_{l}^{4},\mu_{sd}^{5},\mu_{s}^{6},\mu_{sl}^{7},\mu_{v}^{8}\right)$. Afterwards, in iteration *k* of the subgradient optimization procedure, the multiplier vector is updated by **ρ**^k+1^ =**ρ**^k^ + *t^k^g^k^*. The step *t^k^* is determined by $t^{k}=\delta \frac{{Z^{h}}_{IP2}-Z_{D1}(\rho_{k})}{{\left\|g^{k}\right\|}^{2}}$, where $Z_{IP2}^{h}$ is the primal objective function value for a heuristic solution (an upper bound on Z_IP2_), and δ is a constant, 0 < δ ≤ 2.

### 4.3. Getting Primal Feasible Solutions

By applying the Lagrangean Relaxation Method and the Subgradient Method to solve these problems, we can not only determine a theoretical lower bound from the primal feasible solution, but we have also found some helpful hints for the primal feasible solution that are iterated when solving the dual problem.

#### Heuristics for Getting Primal Feasible Solutions

Two stages are introduced in our heuristics for getting the primal feasible solution: the first stage is the multicast routing problem; the second is the capacity assignment and delay calculation.

##### Stage 1: Multicast Routing

There are some hints to be found within the Lagrangean Relaxation Method's Lagrangean Multipliers. In our multicast routing assignment, the set of routing decision variable {*x_p_*}'s corresponding multipliers can be used as each link's arc weight. This way, highly loaded links can be avoided by considering the capacity allocation output from the last iteration's dual problem. Methods such as Dijkstra Algorithm or Prim's minimum spanning tree algorithm, normally used for finding the shortest path, are utilized to find multicast trees for each multicast group. The shortest path algorithm and the complexity of our heuristic can be chosen in stage 1 is *O(*|*N*|*^2^)*. In this stage, the routing algorithm can be summarized as follows:

Step 1: For each group *s*, each link *l*'s arc $\text{weight}=\sum_{d\in D_{s}}\mu_{\text{sdl}}^{1}$, which is obtained from LR problem and presents the degree of importance of each shortest path.
Step 2: Run the Dijkstra algorithm to determine the OD path *p* of each multicast group *s*.

##### Stage 2: Capacity Assignment and Delay Calculation

According to the previous stage, the routing path for each source to reach their destinations can be decided. In this stage, the number of paths passing through the link needs to be calculated in each link's traffic path. Meanwhile, they cannot be violated by Constraint (1.8).

**Figure 10.**
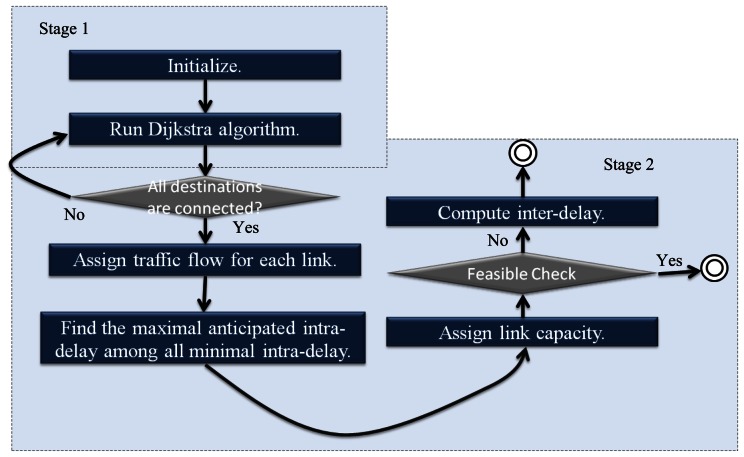
Flow chart of getting primal feasible solution.

Having determined every links' traffic flow, we propose a heuristic which steps are described in Algorithm 1 to find a minimal inter-delay. Through this, each link's allocation capacity can be determined. The time complexity of each iteration is *O(*|*N*|*^2^)*, and below is the pseudo code of our heuristic. A flow chart illustrates the primal feasible solution in Figure 10.

**Algorithm 1.** Capacity assignment and delay calculation.//Input: all links' traffic flow obtained from stage 1//Output: each link's capacity allocation// find anticipated intra-delay for each source-destination path**for** each link *l(i, j)*{ maximal available link capacity for each link =  node capacity * link traffic / sum of the total traffic from the node;}compute each OD path's intra-delay with maximal available capacity;**for** each group *s*{ max_intra_delay = the maximal intra-delay among OD paths;}max_inter_delay = the maximal { max_intra_delay * group's weight;}anticipated intra-delay = max_inter_delay / the belonging group's weight of the path;// assign link capacity per path**for** each path's bottom link *l(i, j)* to source{ adjust capacity(*l(i, j)*, 0);  **if** (each path's anticipated intra-delay!= delay of *l(i, j)* + other link's delay) {  parent = delay of *l(i, j)*;  adjust capacity(*pre_l (i, j)*, parent); }}//compute inter-delay Tinter-delay T = any group's intra-delay * the group's weight;return inter-delay T;

## 5. Experiments and Results

### 5.1. Simulation Environment

In this section, the computational experiments and algorithms are constructed and implemented to analyze the quality of the heuristic being developed. Our experiments are developed in C++, and implemented in a platform with Intel Core2 Quad 2.4 GHz, 1 GB RAM, and Windows Server 2003 Standard with SP2. Table 3 illustrates experiment parameters.

A grid topology is designed with 49 nodes for experiments. To display the characteristics of our proposed algorithm, each source and their corresponding destinations are deployed as far as possible.

In order to compare the performance of LR optimal solution, we propose a simple algorithm, SA, for a simulation to show the benchmark comparison between not optimal, near optimal or optimal solutions. SA procedures of experiments are illustrated in Table 4.

### 5.2. Experiment Results

In our experiments, the solution of the dual problem is defined as *LB*, and the solution of LR based heuristic is defined as *LR*. The solution derived by a simple algorithm is denoted as *SA*. Two performance metrics are utilized to evaluate the solution quality, “Gap” and “Improvement Ratio”. The method of calculating “Gap” and “Improvement Ratio” respectively are shown below:

$$\text{Gap}=\frac{LR-LB}{LR}\times 100\%;$$

Improvement Ratio of $\text{SA}=\frac{SA-LR}{LR}\times 100\%$.

In the following experiment scenario, there are 49 nodes and both source and destination are randomly chosen for each group. Each destination's traffic requirement is also randomly determined. In our experiments, two different dimensions are set for testing our algorithms: number of groups and number of destinations per group. Table 5 shows the result of the experiments under several scenarios:

- The relationship between the number of different destination and minimized end-to-end inter-delay;
- The relationship between the number of different groups for minimized end-to-end inter-delay.

**Figure 11.**
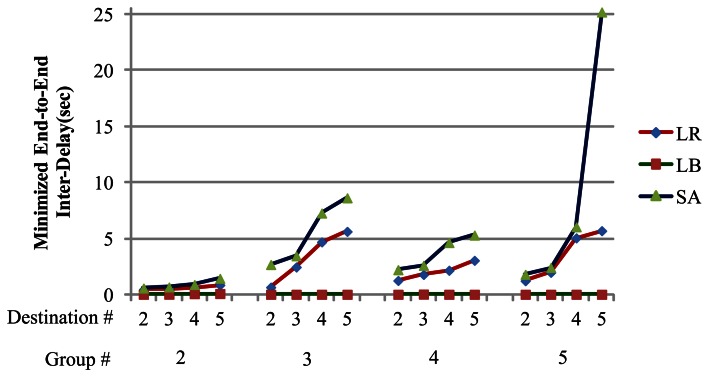
Inter-group end-to-end delay.

Figure 11 indicates that each group can achieve 100% fairness for intra-group and inter-group end-to-end delay. The experiment results can be divided into two parts: the relationship between different number of group for minimized end-to-end inter-delay, and the relationship between different number of destinations and minimized end-to-end inter-delay. A clear overall trend is that when the number of groups increases, the LR and LB algorithms can maintain stability and reduce end-to-end inter-delay, while the SA suffers poor solution quality. At the same time, when the number of destinations per group increases, LR and LB can also maintain stability and reduce end-to-end inter-delay. Based on the experiment results for number of groups and number of destinations, LR and LB ultimately provide superior network performance in terms of perfect fairness in multi-rate multicast wireless sensor networks.

## 6. Conclusions

In our paper, a multicast model of sensor network can be formulated as a “tree forest” type architecture that jointly considers the routing problem and link capacity assignment through various mathematical programming techniques. Fairness can also be achieved in the inter-group and intra-group end-to-end delay in multi-rate multicast wireless sensor network. Our contributions in this research are the solution of an NP-complete problem through mathematical programming techniques to determine the optimal solution LR and LB. Finally, the Lagrangean Relaxation Method and Optimization-based algorithm are provided to solve this problem and have been proven to have good quality after verification with other simple algorithms and LB value. The LR based algorithm and our heuristic are implemented to prove that solution quality is better than that of SA. The solution utilizes the Lagrangean Relaxation Method in conjunction with novel optimization-based heuristics. Computational experiments have been conducted to evaluate the performance of the proposed algorithms. In conclusion, our contribution has been perfectly solving the complicated optimization problem through the Lagrangean Relaxation Method with more efficiency and effectiveness.

## 7. Future Work

The multi-rate multicast wireless sensor network mentioned here is a static environment. For a dynamic case, traffic requirements can be viewed as decision variables. This issue can be addressed by a network administrator that can decide how to efficiently allocate resources to destinations according to the requirements of a dynamic environment. In this case, delay is more sensitive for subscribers. This ultimately results in a tradeoff between delay and fairness. What is the management strategy for addressing fairness and delay perfectly? This will depend on new QoS metric management and control mechanisms.

## Figures and Tables

**Table 1.** Notation descriptions of given parameters.

**Given Parameters**	

**Notation**	**Definition**
*_V_*	The set of all nodes in the network
*_S_*	The set of multicast source nodes
*_Ds_*	The set of destination nodes for each source *s*
*_L_*	The set of all links (*i, j*) in the network, ∀*i*, *j* ∈ *V*, *i* ≠ *j*
*_L_v*	The set of wireless links associated with node *v*, ∀*v* ∈ *V*.
*_P_sd*	The set of paths from source *s* to its destination *d*
*_δ_pl*	1 if link *l* is on the path *p*, and 0 otherwise
*_γ_sd*	The rate of traffic requirement from destination *d* to source *s* (packet/sec)
*_D_sl_(c_sl*,*_g_sl_)_*	The mean delay on link *l* for a multicast tree rooted at source *s*. The delay function is a monotonically increasing and convex function of both link capacity and aggregate flow.
*_d_sl*	The delay on link *l* for a multicast tree rooted at source *s*.
*_β_s*	The degree of importance of a multicast group *s*.
*_H_s*	The minimum hop counting from the farthest destination to the source *s.*
*_I_v*	The incoming links to node *v*, ∀*v* ∈ *V*
*_I_s*	The incoming links to node *s*, ∀*s* ∈ *S*
*_C_v*	The total air interface capacity for node *v*, ∀*v* ∈ *V*

**Table 2.** Notation descriptions of decision variables.

**Decision Variable**	

**Notation**	**Description**
*_x_p*	1 if path *p* is selected for the multicast group *s* to destination *d*, and 0 otherwise.
*_y_sl*	1 if link *l* is in the multicast group *s*, and 0 otherwise.
*_t_sdl*	1 if link *l* is used by destination d of multicast group *s*, and 0 otherwise.
*_c_sl*	The capacity of link *l* in the multicast group *s.*
*_c_l*	The allocated capacity of each link *l*
*_g_sl*	The traffic rate of link *l* in the multicast group *s.*
*_t_s*	The end-to-end delay per path in the multicast group *s.*
*_T_*	The end-to-end delay for each multicast group.

**Table 3.** Experiment environment and parameters.

**Parameter**	**Value**
Topology	Grid network
Number of Nodes	49
Number of groups	2∼5
Range of requested bandwidth	1∼3
Number of destinations in a group	2∼5
Node Capacity	30
Number of iteration	1000
Improvement counter	80
Initial upper bound	0
Initial value of multipliers	0
Test platform	CPU: Intel Core2 Quad 2.4 GHz
	RAM: 1GB RAM
	OS: Windows Server 2003 with SP2
Development tool	Eclipse with g++

**Table 4.** Procedures of simple algorithm.

Step 1.	For group *s*, each link *l*'s arc weight = (1/the sum of its connected nodes' degree)
Step 2.	Run Dijkstra algorithm to determine each OD path *p* of each multicast group *s*.
Step 3.	We assign each link's traffic flow by the destinations whose shortest paths pass the link.
Step 4.	After finding the anticipated intra-delay per path, capacity is allocated for each link. Hence, the objective value can be obtained.

**Table 5.** Experiment result explanation.

**Number of Group**	**Number of Destination**	**LB**	**LR**	**SA**	**Gap (%)**	**I. R. (%)**
2	2	0.025702	0.450961	0.569503	94.3007%	20.8150%
	3	0.026917	0.538237	0.663245	94.9990%	18.8479%
	4	0.075271	0.64264	0.883838	88.2873%	27.2898%
	5	0.08825	0.858677	1.43812	89.7226%	40.2917%
3	2	0.025748	0.65759	2.67525	96.0845%	75.4195%
	3	0.025641	2.45674	3.44923	98.9563%	28.7742%
	4	0.025641	4.6933	7.26076	99.4537%	35.3608%
	5	0.025641	5.62759	8.62115	99.5444%	34.7234%
4	2	0.025641	1.25577	2.21255	97.9581%	43.2433%
	3	0.037905	1.78956	2.60234	97.8819%	31.2327%
	4	0.025641	2.16318	4.63587	98.8147%	53.3382%
	5	0.025641	3.04591	3.29655	99.1582%	7.6031%
5	2	0.025641	1.23	1.79299	97.9154%	31.3995%
	3	0.025641	1.98478	2.40776	98.7081%	17.5674%
	4	0.025641	5.05017	6.04086	99.4923%	16.3998%
	5	0.025641	5.69069	25.1829	99.5494%	77.4026%
